# Long-term posterolateral spinal fusion in rabbits induced by rhBMP6 applied in autologous blood coagulum with synthetic ceramics

**DOI:** 10.1038/s41598-022-14931-2

**Published:** 2022-07-08

**Authors:** Nikola Stokovic, Natalia Ivanjko, Marko Pecin, Igor Erjavec, Ana Smajlović, Marina Milesevic, Sven Karlovic, Hrvoje Capak, Zoran Vrbanac, Drazen Maticic, Slobodan Vukicevic

**Affiliations:** 1 Laboratory for Mineralized Tissues, Center for Translational and Clinical Research, School of Medicine, University of Zagreb, Salata 11, 10000 Zagreb, Croatia; 2 Scientific Center of Excellence for Reproductive and Regenerative Medicine, Zagreb, Croatia; 3 Clinics for Surgery, Orthopedics and Ophthalmology, School of Veterinary Medicine, University of Zagreb, Zagreb, Croatia; 4 Faculty of Food Technology and Biotechnology, University of Zagreb, Zagreb, Croatia; 5 Department of Radiology, Ultrasound Diagnostics and Physical Therapy, Faculty of Veterinary Medicine, University of Zagreb, Zagreb, Croatia

**Keywords:** Drug discovery, Recombinant protein therapy, Preclinical research

## Abstract

Autologous bone graft substitute (ABGS) containing rhBMP6 in autologous blood coagulum (Osteogrow) is a novel therapeutic solution for bone regeneration. This study is aimed to investigate the long-term outcome of ABGS with synthetic ceramics (Osteogrow-C) in rabbit posterolateral spinal fusion (PLF) model. Osteogrow-C implants were implanted bilaterally between rabbit lumbar transverse processes. We compared the outcome following implantation of ABGS with ceramic particles of different chemical composition (TCP and biphasic ceramics containing both TCP and HA) and size (500–1700 µm and 74–420 µm). Outcome was analyzed after 14 and 27 weeks by microCT, histology, and biomechanical analyses. Successful bilateral spinal fusion was observed in all animals at the end of observation period. Chemical composition of ceramic particles has impact on the PLF outcome via resorption of TCP ceramics, while ceramics containing HA were only partially resorbed. Moreover, persistence of ceramic particles subsequently resulted with an increased bone volume in implants with small particles containing high proportion of HA. ABGS (rhBMP6/ABC) with various synthetic ceramic particles promoted spinal fusion in rabbits. This is the first presentation of BMP-mediated ectopic bone formation in rabbit PLF model with radiological, histological, and biomechanical features over a time course of up to 27 weeks.

## Introduction

Posterolateral spinal fusion (PLF) is a surgical procedure to treat degenerative spine conditions, usually caused by aging, tumors, infections or arthritis, and include degenerative disc disease, spinal instability, spondylolisthesis, and symptomatic scoliosis^1–4^. In this procedure, osteoinductive implants and materials are placed between transverse processes to achieve bridgement and subsequently form a new load-bearing segment in the spine. Due to its inherent osteoinductive, osteoconductive, and osteogenic properties, autologous bone graft (ABG) harvested from the iliac bone crest is considered the gold standard for PLF. Moreover, autograft for revision surfery might be obtained from femur employing Reamer-Irrigator-Aspirator (RIA) technique^5–7^. However, the use of ABG inevitably has several disadvantages associated with donor site morbidity, including pain, wound infection, skin scarring, and deformity, with a prolonged time of surgical procedure and increased blood loss^2,8–10^.

To overcome ABG limitations, different autologous bone graft substitutes (ABGS) containing bone morphogenetic proteins (rhBMP2 and rhBMP7) on various carriers, including natural polymers, synthetic polymers, ceramics, and combinations of these materials^11–16^, have been tested in rabbits^3,4,17–25^, sheep^1,26^ and non-human primates in the PLF indication^8,21,27^. Moreover, osteoinductive devices containing rhBMP2 combined with bovine collagen and ceramics have been evaluated in human spine PLF clinical trials but did not obtain market approval^2,9,28^. Despite that, large dose rhBMP2 based bovine collagen device has been commonly used *off-label* in PLF indication^29^ resulting in immunogenicity of the collagen carrier, postoperative inflammation, radiculopathy, heterotopic ossification, vertebral bone resorption and retrograde ejaculation^30–32^.

ABGS containing rhBMP6 within autologous blood coagulum (ABC) named Osteogrow is a novel therapeutic solution for various clinical indications, including spinal fusions and segmental bone defect management evaluated both in preclinical^33–41^ and clinical studies^42–44^. BMP6 is more potent than BMP2 and BMP7 in promoting osteoblast differentiation in vitro and promoting bone regeneration in vivo due to its resistance to Noggin^45,46^. ABC is a physiological native BMP carrier which suppresses foreign body response, promotes BMP binding to plasma proteins within the fibrin meshwork to allow a sustained in vitro BMP release^33,34,36^.

We have tested the potential use of this novel ABGS in PLF preclinical studies. To enhance the biomechanical properties of Osteogrow implants, we added compression resistant matrix (CRM) to implants^34^. First, we evaluated the use of ABGS with allograft (Osteogrow-A) in rabbit and sheep PLF models^34,35^, and following the successful outcome, a similar ABGS formulation was tested in patients undergoing PLF due to degenerative disc disease (EudraCT number 2017-000860-14). However, allograft bone has limitations, including regulatory issues, immunogenicity and low but still existing viral transmission risk^4,47^. Therefore, to find a safe and efficient alternative to allograft, we demonstrated that ABGS with synthetic calcium phosphate (CaP) ceramic particles (Osteogrow-C) promoted successful PLF in rabbits seven weeks following surgery^37^. CaP ceramics include tricalcium phosphate (TCP; Ca_3_PO_4_) and hidroxyapatite (HA; Ca_10_(PO_4_)_6_(OH)_2_). The main difference among them is resorbability since TCP is resorbed significantly faster than HA. Moreover, TCP and HA might be combined in various ratios into biphasic calcium phosphate (BCP) to adjust ceramics resorbability^48,49^. Due to a single time point and a short observation period, in our previous work^37^ we could not determine the longevity of newly formed bone and the influence of the chemical composition of ceramics (TCP vs BCP) on the spinal fusion outcome over a prolonged period of time.

In the present study, we explored the influence of chemical composition, size of ceramic particles, and the BMP6 dosing on the outcome of PLF spinal fusion in rabbits. We also described the time course of ectopic bone induction between the transverse processes and determined bone volume and biomechanical properties of newly induced ectopic bone.

## Results

### Spinal fusion success rate and segmental mobility testing

**Figure 1:**
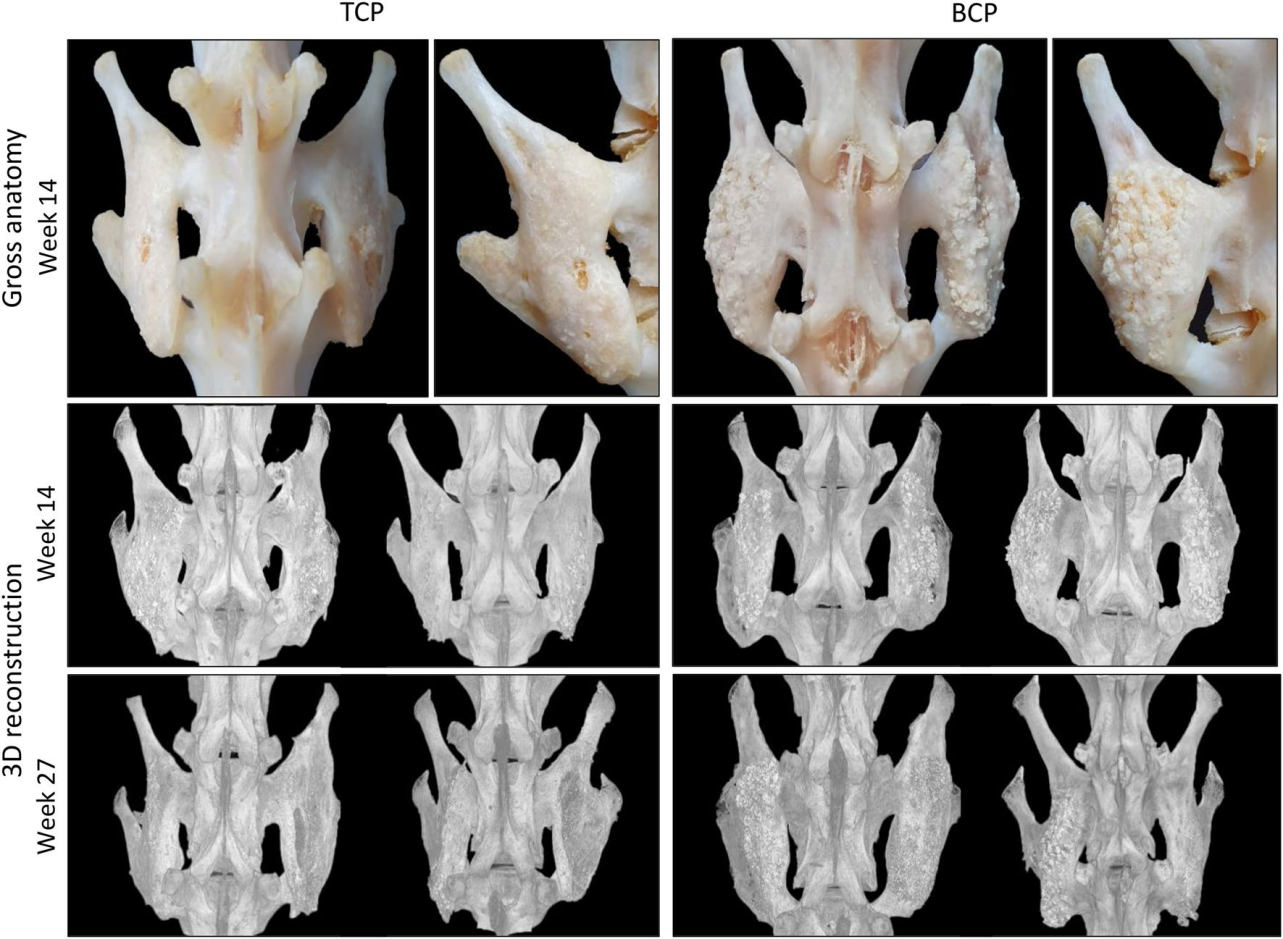
Gross anatomy and microCT 3D reconstruction of newly induced ectopic bone fused with transverse processes. ABGS implants with TCP (left) and BCP (right) ceramics induced ectopic bone which fused with adjacent transverse processes as observed on macerated specimens 14 weeks following implantation (1st row) and on microCT 3D reconstruction 14 weeks (2nd row) and 27 weeks (3rd row) after surgery. In animals with follow-up period of 14 weeks, the spinal fusion was achieved with ABGS (rhBMP6/ABC) and 500–1700 µm TCP or BCP ceramics with the rhBMP6 dose of 125 (left side of the spine) or 250 µg (right side of the spine) (n = 4 per group). In animals after 27 weeks, the spinal fusion was achieved with ABGS containing 125 µg rhBMP6 within ABC with TCP or BCP ceramics in two different particle sizes: 500–1700 µm (left side of each animal) or 74–420 µm (right side of each animal) (n = 6 per group).

**Figure 2:**
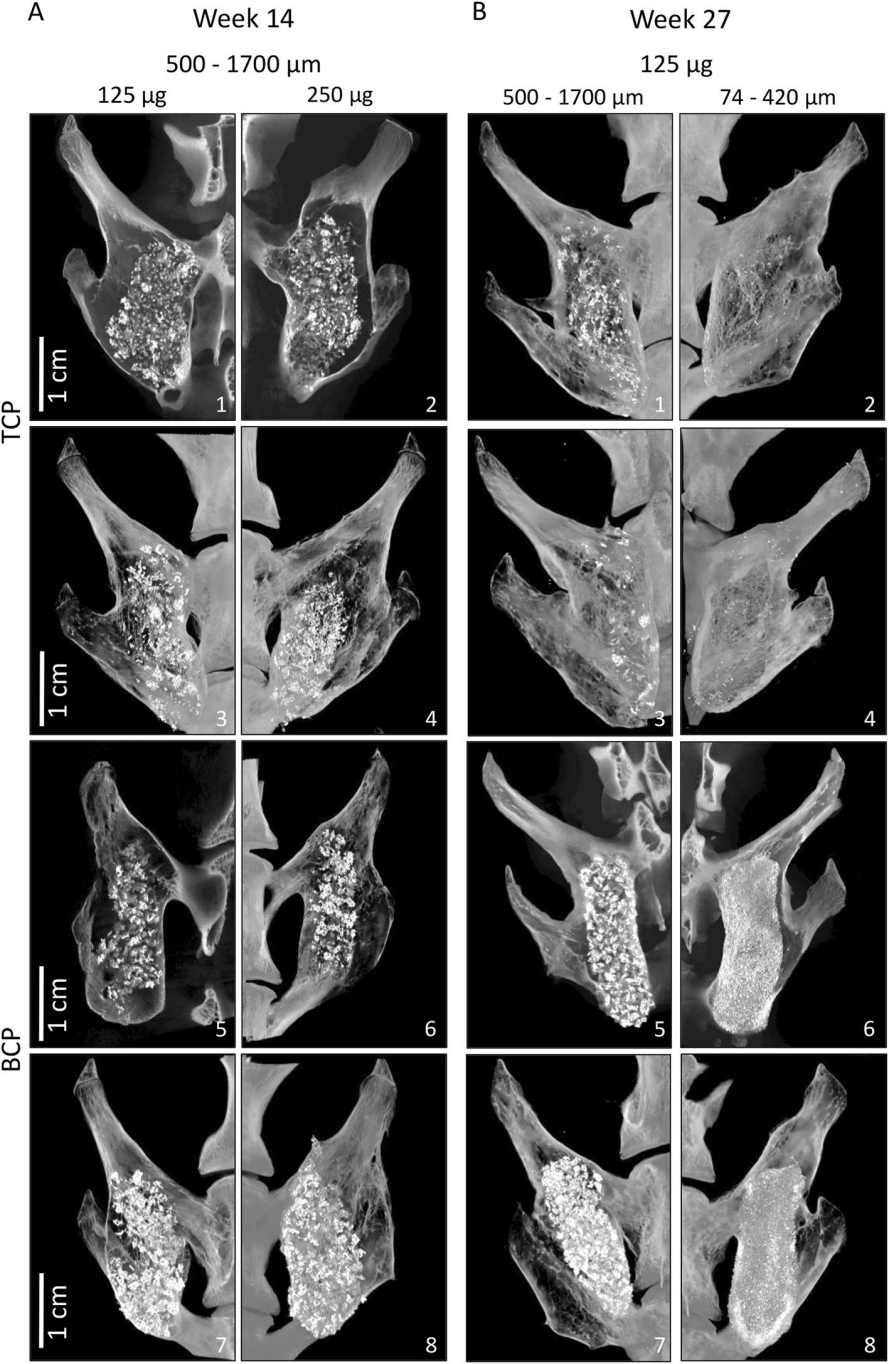
MicroCT sections through newly induced ectopic bone fused with transverse processes. Different ABGS formulations with synthetic ceramics successfully induced the fusion between adjacent transverse processes as shown in eight rabbits. (**A**) Spinal fusion after 14 weeks induced by ABGS containing 500–1700 µm TCP or BCP (TCP/HA 80/20) ceramic particles with the rhBMP6 dose of 125 µg (1, 3, 5, 7) or 250 µg (2, 4, 6, 8) (n = 4 per group). (**B**) Spinal fusion after 27 weeks induced by ABGS containing TCP or BCP (TCP/HA 40/60) particles in two different sizes; 500–1700 µm (1, 3, 5, 7) and 74–420 µm (2, 4, 6, 8) with 125 µg rhBMP6 (n = 6 per group). Scale bars are indicated in lower left corner.

All tested ABGS implants with ceramic particles induced new bone and bilateral bridgement between adjacent transverse processes as observed at the end of weeks 14 and 27 by microCT 3D reconstructions and gross anatomy specimens observation (Fig. 1). Segmental immobility by palpatory testing was observed in all specimens (16/16 in the first and 24/24 in the second experiment). Moreover, integration of the new bone with transverse processes was confirmed on microCT sections (Fig. 2).

### MicroCT analyses

**Figure 3:**
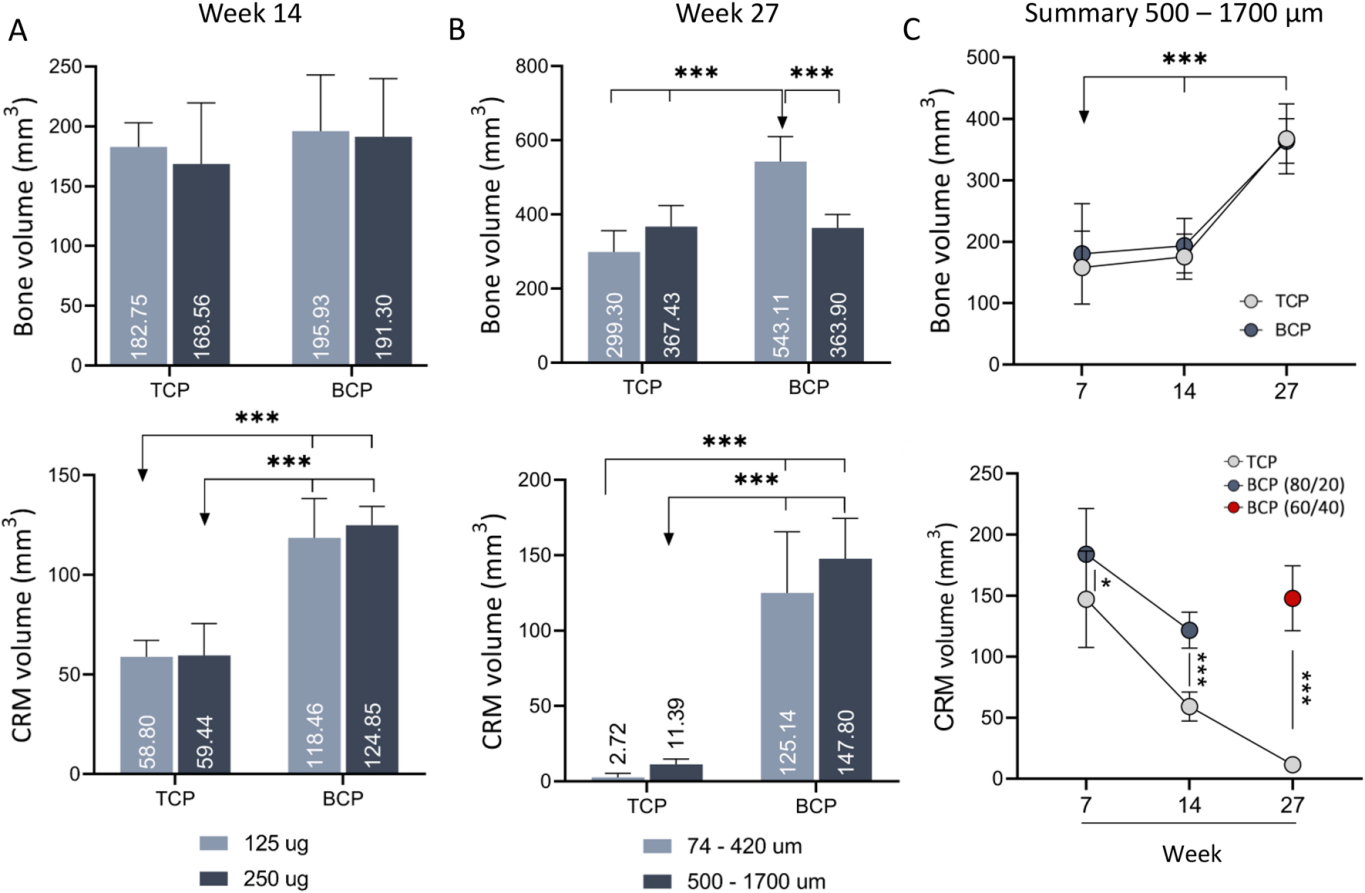
MicroCT analyses of newly induced ectopic bone fused with transverse processes. (**A**) Bone volume (1st row) and CRM volume (2nd row) in specimens containing TCP and BCP ceramic particles 14 weeks following ABGS implantation. Bars represent implants with 125 µg (light blue) and 250 µg (dark blue) rhBMP6 (n = 4 per group). (**B**) Bone volume (1st row) and CRM volume (2nd row) in specimens with 74–420 µm (light blue) and 500–1700 µm (dark blue) TCP or BCP ceramic particles 27 weeks following ABGS implantation (n = 6 per group). (**C**) Summarized values for bone volume (1st row) and CRM volume (2nd row) in specimens containing TCP or BCP in medium size range particles (500 to 1700 µm) after 14 and 27 weeks as compared to our previously published work with a follow-up period of 7 weeks (modified from^37^). All P values below 0.05 were considered significant and are marked with asterisks: *(P ≤  0.05), **(P ≤  0.01), ***(P ≤  0.001).

After 14 weeks, the amount of newly formed bone was extensive in all animals (Fig. 3, first row). The differences among experimental groups were not significant neither regarding the chemical composition of ceramic particles (TCP vs. BCP (TCP/HA 80/20)) nor the rhBMP6 dose (125 µg vs. 250 µg) (Fig. 3A, first row). After 27 weeks, the bone volume was maintained in all specimens. However, bone volume was significantly higher in implants containing small BCP (TCP/HA 40/60) particles comparing to other experimental groups (Fig. 3B, first row).

At week 14 following surgery, the CRM volume was significantly decreased in groups containing TCP than in specimens with BCP (TCP/HA 80/20) (Fig. 3A, second row). At week 27, the differences in CRM volume containing particles of different chemical composition (TCP and BCP (TCP/HA 40/60)) were more pronounced due to a longer follow-up period and increased proportion of HA in biphasic ceramic particles (Fig. 3B, second row).

To establish the timeline of ectopic bone formation at the ectopic site in the rabbit PLF model, we also compared the results from this study to data from the previous study^37^ (Fig. 3C). Since there were no significant differences in bone volume among different experimental groups at observed time points, we pooled all the samples with similar particle size (500–1700 µm) and same rhBMP6 dose (125 μg) from the same time point. The CRM volume was shown separately for TCP and BCP ceramics (500–1700 µm). This analyses revealed that the amount of bone increased through the follow-up of 7, 14, and 27 weeks (Fig. 3C, first row), while the CRM volume decreased in time, and this was especially pronounced in particles containing TCP alone (Fig. 3C, second row).

### Histological analyses

**Figure 4:**
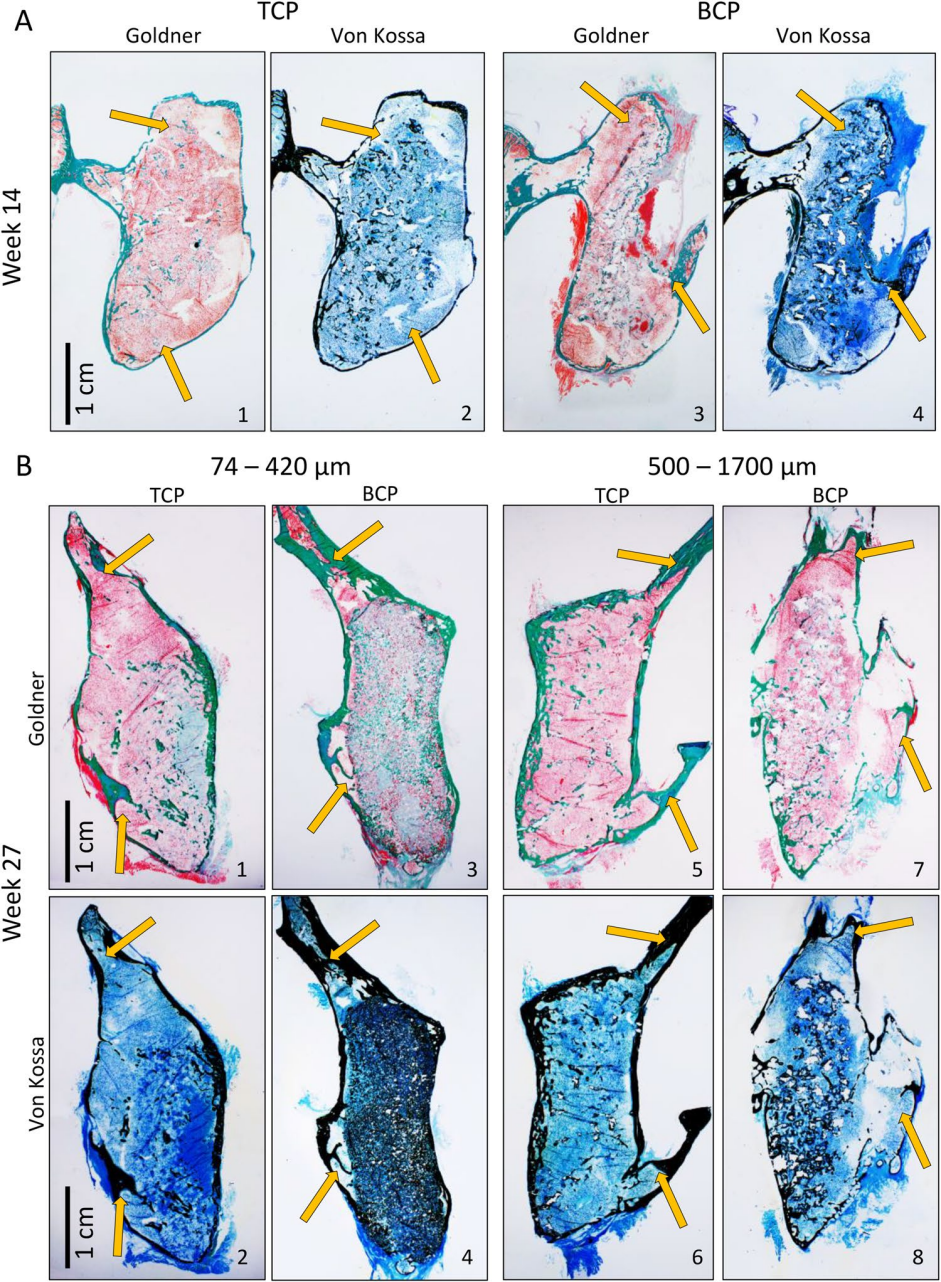
Undemineralized histological sections of newly formed bone fused with adjacent transverse processes on week 14 and 27 following ABGS implantation. New bone induced by ABGS fused with adjacent transverse processes. Cortical bone formed continuity with transverse processes (yellow arrows). (**A**) On week 14, TCP (1–2) appeared more resorbed than BCP (TCP/HA 80/20) (3–4). (**B**) 27 weeks after implantation the differences between TCP and BCP (TCP/HA 40/60) were pronounced: namely, TCP particles were almost completely resorbed (1, 2, 5, 6) while BCP particles were slightly resorbed (3, 4, 7, 8). Histological sections were stained with Goldner or Von Kossa stain. Scale bars are indicated in the lower left corner.

Successful integration of the newly formed bone with adjacent transverse processes was observed in all specimens obtained after 14 and 27 weeks following ABGS implantation (Fig. 4).

Histological analyses of specimens obtained on week 14 revealed that new bone contained pronounced cortical bone, which was in continuity with the cortical bone of the transverse processes (Fig. 4A). Moreover, new bone was present on the surfaces and between ceramic particles (Fig. 4A). Bone trabeculae were surrounded with bone marrow containing a large amount of both adipocytes and hematopoietic cells. Importantly, ceramic particles as well were fully integrated with the transverse processes. Notably, there was no significant difference in the bone volume and CRM volume regardless of the BMP dose.

**Figure 5:**
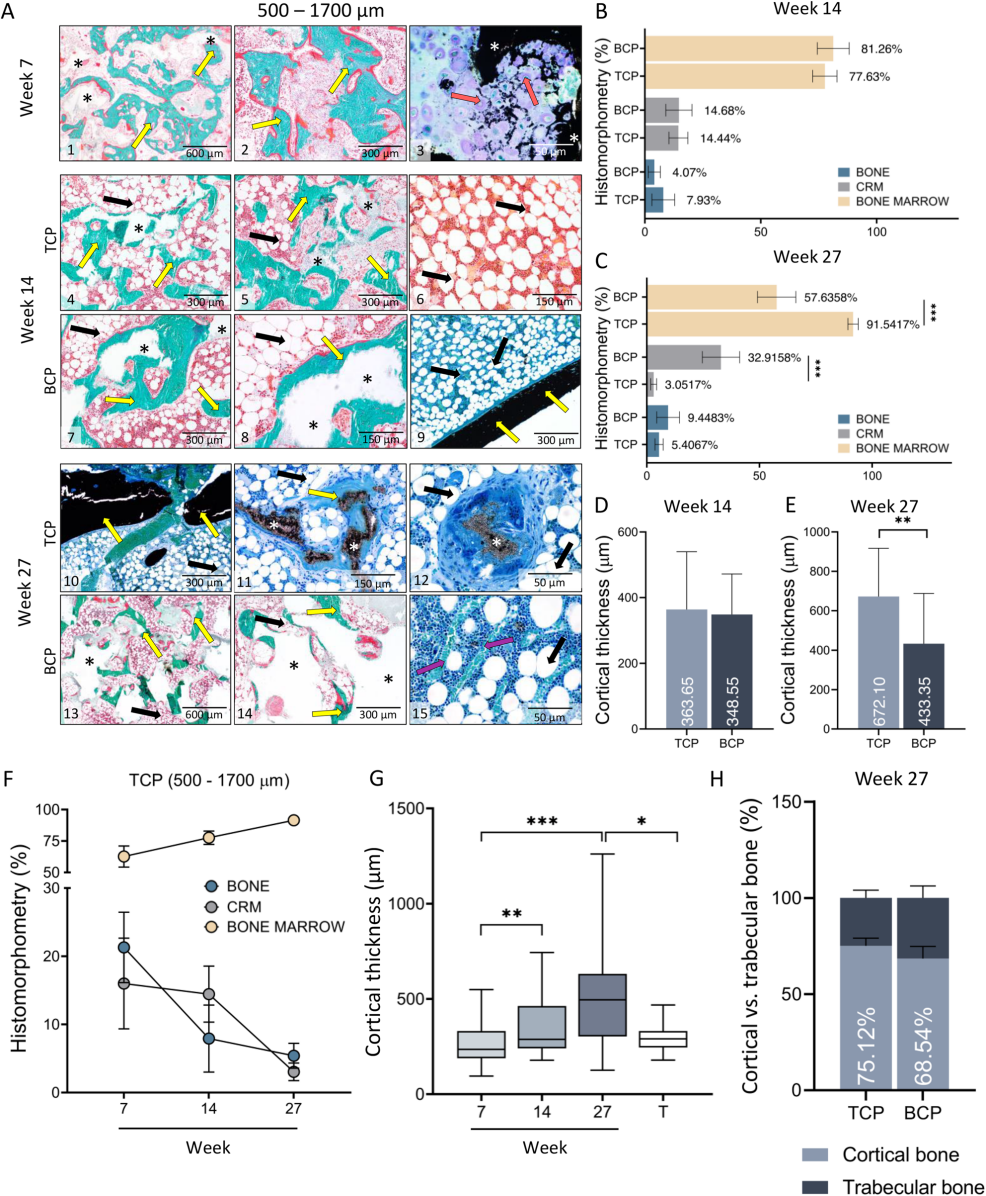
Histological analysis of newly formed bone fused with adjacent transverse processes on week 7, 14 and 27 following implantation of ABGS containing 500–1700 µm particles. (**A**) Newly formed bone is induced by ABGS at the surfaces and between synthetic ceramic particles (**A1–2**). Small areas with ongoing endochondral ossification were present in few specimens (**A3**) (unpublished data from Stokovic N et al., Bone^37^). 14 weeks following implantation newly formed bone surrounded with abundant bone marrow was present between ceramic particles (**A4–8**). TCP particles (**A4–5**) were more resorbed compared to BCP (TCP/HA 80/20) particles (**A7–8**). Bone marrow contained both hematopoietic cells and adipocytes (**A6**). Cortical bone was present at the boundaries (**A9**). Number of analysed specimens was 4 per group. 27 weeks after surgery TCP particles are significantly resorbed and the most important structural feature is pronounced cortical bone. Remnants of the TCP ceramic particles surrounded with abundant bone marrow were visible in the central portion of the implant (**A10–12**). In contrast, BCP (TCP/HA 40/60) particles were unresorbed and bone was present on the surfaces and between the particles (**A13–14**) Bone marrow contained both hematopoietic cells and adipocytes (**A15**). Sections were stained by Goldner (**A1–2, A4–8, A13–14**) or Von Kossa (**A3, A9–12, A15**) stain. Yellow arrows indicated newly formed bone, asterisks ceramic particles, orange arrows endochondral ossification and purple arrows blood vessels. Number of analysed specimens was 6 per group. Scale bars are indicated in the right corner of each image. Proportions (%) of bone, CRM and bone marrow among groups with 500–1700 µm TCP or BCP particles on week 14 (**B**) and 27 (**C**). Cortical thickness on weeks 14 (**D**) and 27 (**E**) of new bone induced by ABGS containing 500–1700 µm TCP and BCP particles. Results on week 14 are presented regardless of the applied rhBMP6 dose (**B,D**). (**F**) Summarized values (TCP, 500–1700 µm) of bone, CRM and bone marrow in the central part of the newly formed bone on 7th (modified or unpublished data from^37^), 14th and 27th week. (**G**) Cortical thickness of implants through 7th, 14th and 27th week; results are gathered from all groups from specified time point. Thickness of transverse processes (T) was used as a control. (H) MicroCT determined proportions (%) of cortical and trabecular bone among experimental groups on week 27. All P values below 0.05 were considered significant and are marked with asterisks: *(P ≤ 0.05), **(P ≤ 0.01), ***(P ≤ 0.001).

**Figure 6:**
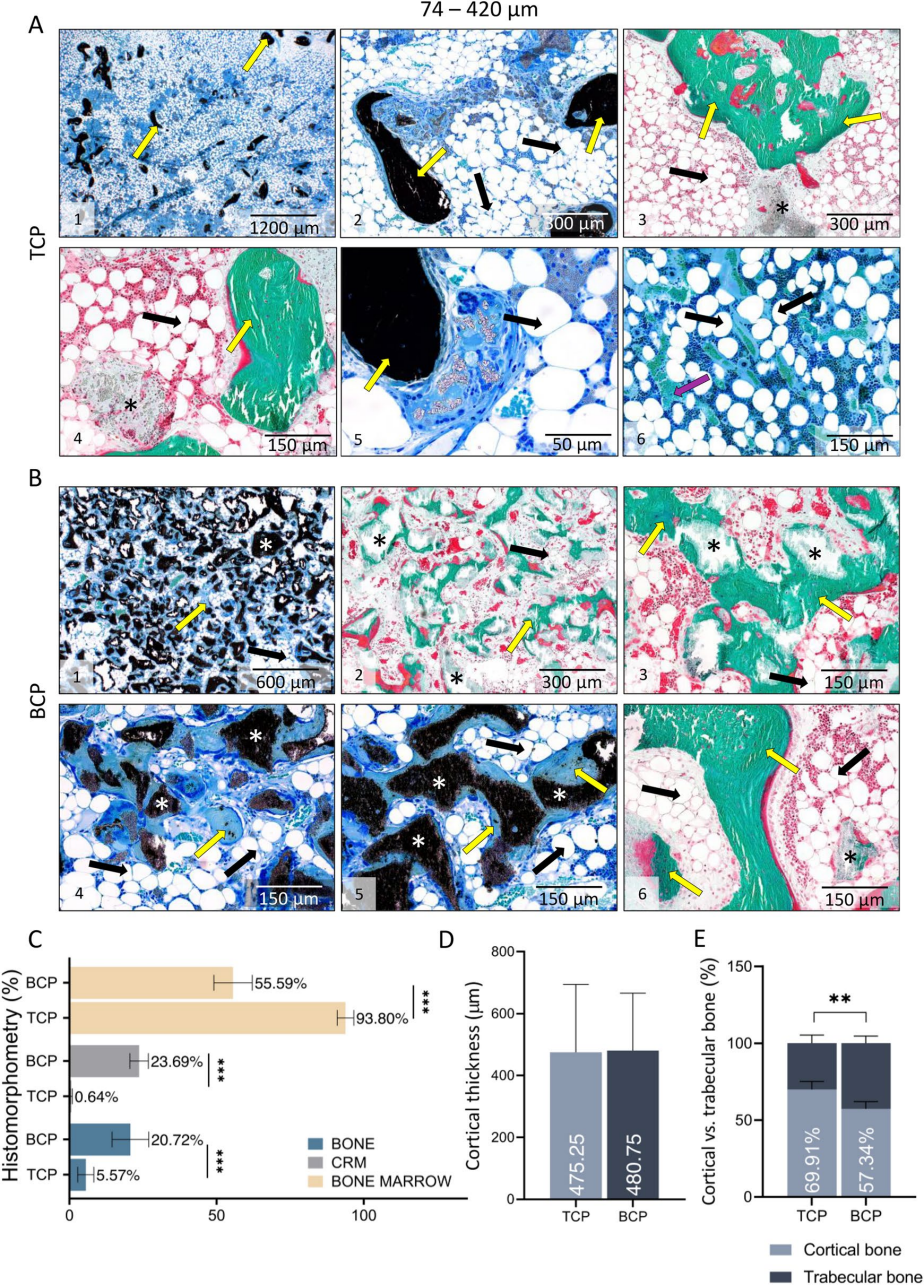
Histological analysis of newly formed bone fused with adjacent transverse processes on week 27 following implantation of ABGS containing 74–420 µm particles. The difference in histological feature of specimens with TCP (**A**) and BCP (TCP/HA 40/60) (**B**) particles 27 weeks after implantation was significant: in specimens with TCP particles (**A1–6**), ceramics was almost completely resorbed and the main histological features were bone trabeculae with abundant bone marrow. Only remnants of ceramic particles were observed. On the contrary, in specimens with BCP ceramics (**B1–6**) ceramic particles were unresorbed and there was a dense bone network between and on the surfaces of ceramic particles. Sections were stained by Goldner (**A3–4, B2–3, B6**) or Von Kossa (**A1–2, A5–6, B1, B4–5**) stain. Yellow arrows indicated newly formed bone, asterisks ceramic particles, and purple arrows blood vessels. Number of analysed specimens was 6 per group. Scale bars are indicated in the right corner of each image. Scale bars are indicated in the right corner of each image. **(C)** Proportions (%) of bone, CRM and bone marrow. **(D)** Cortical thickness among groups with 74–420 µm TCP and BCP ceramics on week 27 after implantation. **(E)** MicroCT determined proportions (%) of cortical and trabecular bone among experimental groups on week 27. All P values below 0.05 were considered significant and are marked with asterisks: *(P ≤ 0.05), **(P ≤ 0.01), ***(P ≤ 0.001).

In specimens from week 27 there was a striking difference among experimental groups with TCP and BCP particles containing TCP and HA in 40/60 ratio (Figs. 5A, 6A,B). TCP particles were almost completely resorbed while BCP particles were persistent over time. Specimens with TCP ceramics contained bone trabeculae, abundant bone marrow and remnants of the TCP particles. Importantly, 500–1700 µm particles were less resorbed when compared to 74–420 µm particles. In contrast, specimens with BCP particles contained persistent ceramic particles, bone on the surfaces and between the particles. Important histological feature of all specimens was a cortical bone continuity with transverse processes (Fig. 4B). Similar to findings on week 14, bone marrow contained both hematopoietic cells and adipocytes.

### Histomorphometric analyses

Histomorphometric analyses were conducted to determine the proportion of bone, bone marrow, and CRM in the central part of bone fused with transverse processes as well as thickness of the cortical bone.

On week 14 the differences in measured parameters were not significant among experimental groups (Fig. 5B). However, on week 27, the proportion of CRM and bone were higher, while proportion of bone marrow was lower in specimens containing BCP than TCP ceramics (Figs. 5C, 6C). Cortical bone thickness on week 27 was slightly increased in specimens with 500–1700 µm TCP ceramics (Figs. 5E, 6D), while thickness among experimental groups on week 14 was comparable (Fig. 5D). Moreover, microCT analyses revealed that on week 27 cortical bone formed the majority of total bone volume (Figs. 5H, 6E).

Obtained results were retrospectively compared with the findings at week 7 from our previous work (Fig. 5F,G). Histomorphometric analyses revealed that the amount of bone, bone marrow, and CRM were comparable on weeks 14 and 27. However, on week 27, the amount of bone and CRM were significantly decreased while the amount of bone marrow was significantly increased as compared to week 7 (Fig. 5F). On the contrary, the thickness of the cortical bone was significantly higher on week 27 and 14 when compared to week 7 (Fig. 5G).

### Biomechanical testing

Biomechanical properties of newly formed bone integrated with adjacent transverse processes obtained on weeks 14 and 27 were superior to native transverse processes.

**Figure 7:**
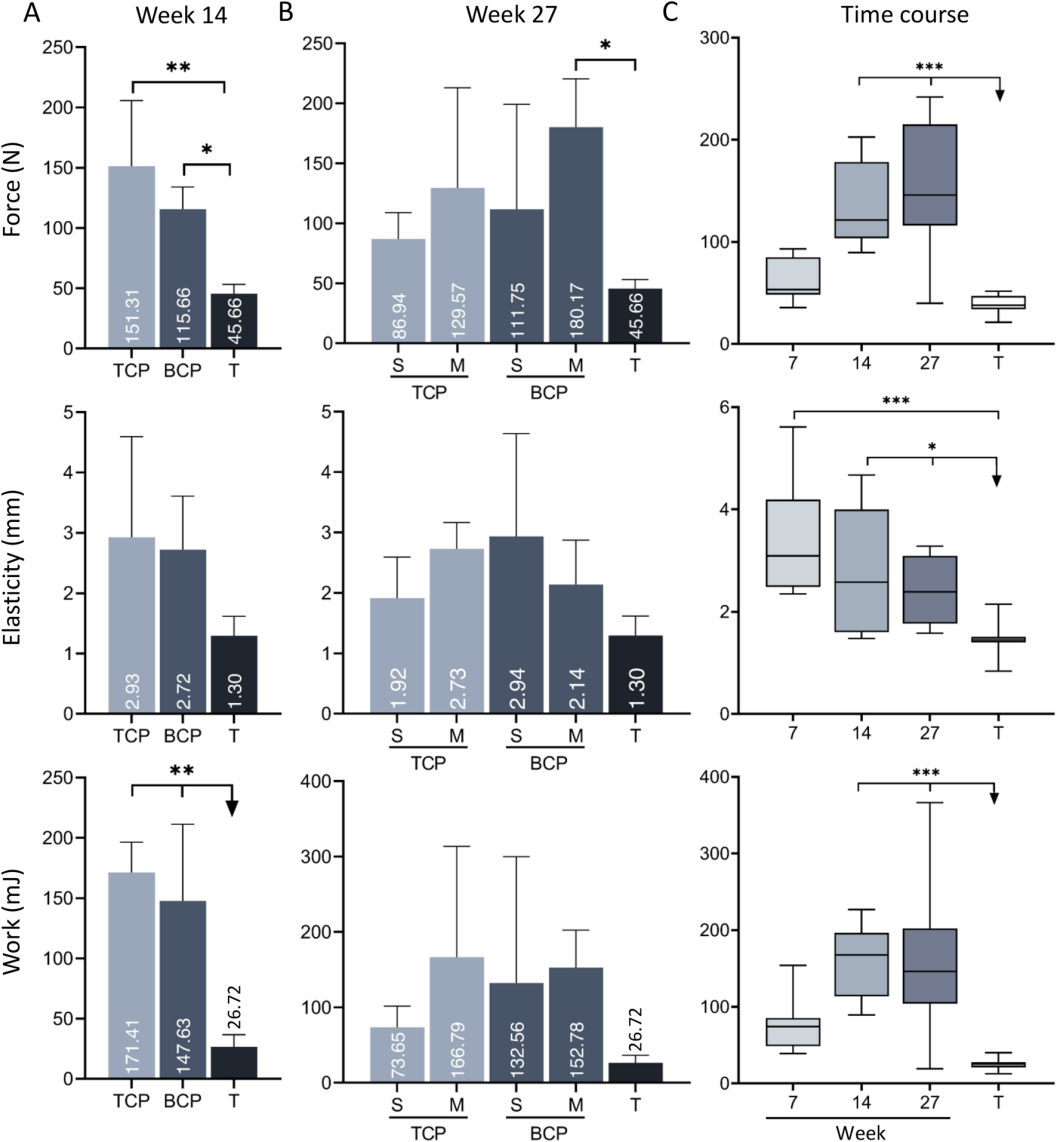
Biomechanical properties of newly formed bone fused with adjacent transverse processes. **(A)** Biomechanical properties (force, elasticity and work-to-break) of specimens containing TCP or BCP (500–1700 µm) ceramic particles on week 14 following implantation. Specimens were grouped according to the chemical composition of particles regardless of the rhBMP6 dose. Transverse processes (T) were used as a control group. **(B)** Biomechanical properties of specimens containing TCP or BCP ceramic particles 27 weeks following ABGS implantation. The size of particles was either 500–1700 µm (medium, M) or 74–420 µm (small, S). Transverse processes (T) were used as a control group. Number of analysed specimens was 3–4 per group. **(C)** Summarized values for force, elasticity and work-to break after 14 and 27 weeks and our previous result at 7 weeks after implantation (modified from^37^). *T* transverse processes. All P values below 0.05 were considered significant and are marked with asterisks: *(P ≤ 0.05), **(P ≤ 0.01), ***(P ≤ 0.001).

Specimens from 14 weeks were grouped based on the CRM chemical composition regardless of the rhBMP6 dose (Fig. 7, first column), and there were no differences in observed biomechanical parameters (force, elasticity, and work) among specimens with TCP and BCP (TCP/HA 80/20).

After 27 weeks, we compared biomechanical parameters of specimens containing medium (500–1700 µm) and small (74–420 µm) particles with two different chemical compositions (TCP and BCP (TCP/HA 40/60)) (Fig. 7, second column). Specimens containing medium BCP particles required significantly higher force and work to break the fusion than specimens containing small TCP particles.

Finally, biomechanical properties of bone obtained on weeks 14 and 27 in this study were compared with the results of our previous study in which biomechanical testing was conducted seven weeks after implantation^37^. Force and work needed to break the bone on weeks 14 and 27 were significantly higher than on week 7 following implantation (Fig. 7, third column). On the other hand, the elasticity of new ectopic bone on week 7 was higher than on week 14 and 27.

## Discussion

Finding an optimal autologous bone graft substitute for posterolateral spinal fusion is a challenging task in bone tissue engineering due to the relatively large distance between transverse processes, which should be bridged with newly induced ectopic bone, presence of compressive forces in the environment and vicinity of critical anatomical structures. Bone morphogenetic proteins are potent osteoinductive molecules and prerequisite for ectopic bone formation^50–52^. However, they require a carrier that will sustain the protein at the treatment site^11–16,53^. To address this issue, several BMP carrier/delivery systems have been proposed and tested in the PLF model: collagen^4,19,22^, synthetic polymers^25,54^, calcium phosphate (CaP) ceramics^3,18,21,22,24,26,27,55^ and combination of collagen and CaP ceramics^1,8,18,21^ or synthetic polymers and CaP ceramics^23^. However, up to the present, none of these devices has been clinically tested and approved for clinical use.

ABGS comprised of rhBMP6 within autologous blood coagulum as a physiological BMP carrier and synthetic ceramics as a compression resistant matrix is a novel osteoinductive device for bone regeneration^33–35,37^. In our previous studies on rat subcutaneous model we addressed several unresolved issues regarding ABGS formulation, including the optimal dose and method of rhBMP6 application as well as the optimal combination of size and chemical composition of ceramic particles^38,41,56^. Moreover, in our first PLF study with ceramics as CRM we have demonstrated that ABGS containing synthetic ceramics named Osteogrow-C promotes spinal fusion and confirmed the finding from rat studies that there are two equally efficient methods of BMP6 application: rhBMP6 might be lyophilized on ceramic particles or directly added to the autologous blood^37,56^. However, due to the relatively short follow-up period (7 weeks), we were unable to observe the effect of the chemical composition of particles on the CRM resorption rate. In the present study, for the first time, we investigated how both chemical composition and particle size of ceramics affect the outcome in the rabbit PLF model over a prolonged period of time.

First, to determine the effect of chemical composition on the PLF outcome we conducted an experiment with a prolonged follow-up period of 14 weeks. In this experiment, the resorption rate of TCP particles was significantly higher than that of BCP particles. However, the amount of bone and biomechanical properties of the new ectopic bone were comparable between specimens containing TCP and BCP (TCP/HA 80/20) particles. Moreover, we showed that 125 µg per implant was sufficient, and there was no further increase in bone volume with an additional amount of rhBMP6 which is in line with previous studies using rhBMP2 on collagen or ceramics as a carrier^3,4,24^. Second, to further explore the influence of CRM chemical composition on the outcome and longevity of bone in the PLF model, we conducted another experiment in which we prolonged the follow-up period to 27 weeks and increased the proportion of HA in BCP (to TCP/HA 40/60). Moreover, we compared the PLF outcome between ABGS formulations with medium and small particles in this experiment. The main finding was that different chemical composition of particles, regardless of their particle size, might result in large difference in the amount of CRM after an extended time period. Moreover, the volume of newly formed bone was increased in implants containing small biphasic ceramic particles which were unresorbed in time. We believe that chemical composition of ceramics might be preferred according to the clinical indication. For instance, highly resorbable TCP might be used for indications in which Osteogrow C is used to restore bone defect and where newly formed bone should mimic native bone. On the other hand, biphasic ceramics might be preffered in indication such as PLF where unresorbed ceramic particles and increased bone volume might enhance biomechanical properties of the fusion mass over a long period of time. However, it is essential to emphasize that all tested ABGS formulations in this study resulted in successful spinal fusion with significant biomechanical properties. This was the first time that ceramics which differed in both size and chemical composition were compared in the PLF animal model since the majority of previous studies evaluated only one type of ceramics^3,20,23,24,26^ with different BMP doses or compared them to autograft. Only few PLF studies compared ceramics with different TCP/HA ratios; however, with a short follow-up period of only 5 weeks differences among groups could not have been observed^21^.

The other major shortcoming of previously published PLF studies was the relatively short follow-up period which was typically shorter than eight weeks^3,17,18,20–24,37,57–59^. Although evaluation at these time points was sufficient for determining spinal fusion outcome, the longevity of ectopic bone was not followed. Longevity of bone in rabbit PLF studies was evaluated only in few studies with a follow-up period of 14 weeks or longer^4,34^. However, these studies had only one observation time point, which did not allow to establish a time course of bone formation and remodeling in the rabbit PLF model since follow-up was limited only to x-ray screening of the result outcome. Completion of the aforementioned series of PLF experiments allowed us for the first time to determine the timeline of ectopic bone formation in the rabbit PLF model and to describe how radiological, histological, and biomechanical findings change in time. MicroCT analyses revealed that the overall amount of bone increased from week seven till week 27. This was in accordance with a previously published study in which bone volume was determined on a series of x-ray images through the follow-up period of 24 weeks^4^. Increase of bone volume in time might be attributed to increase of cortical thickness and increased mineralization of bone over the period of 27 weeks. Moreover, histological analyses revealed that the structure of newly formed bone changed significantly: on week 7, there was more bone between the particles with a discrete cortical bone while at later time point (week 14 and 27), cortical bone was more pronounced with a decreased amount of bone between the particles. These microCT and histological findings correlated with the improved bone biomechanical properties on weeks 14 and 27 as compared to week 7.

## Conclusions

ABGS containing rhBMP6 in ABC with different ceramic particles promoted bilateral spinal fusion in the rabbit PLF model. Successful spinal fusion and integration of the newly formed bone with adjacent transverse processes were observed in all implanted specimens by microCT, histological sections and by biomechanical testing. In the long-term follow-up of 27 weeks, the most significant difference between experimental groups was seen in implants with a different chemical composition of ceramics because TCP was resorbed, while ceramics containing HA were only partially resorbed. Importantly, persistence of ceramic particles resulted with the increased bone volume in implants containing small, biphasic ceramic particles which were unresorbed in time.

## Material and methods

### Experimental design

First, we compared the outcome of PLF 14 weeks following implantation of ABGS (rhBMP6/ABC) containing synthetic ceramic (500–1700 µm) particles in two different chemical compositions (TCP and BCP with TCP/HA in 80/20 ratio) and two different rhBMP6 doses (125 µg and 250 µg/per implant). Based on the obtained results, we designed another experiment in which we prolonged the follow-up period to 27 weeks and increased the proportion of HA in BCP (to TCP/HA 40/60) to achieve more pronounced difference between residual ceramics and to determine whether preferred outcome is ceramics resorption or preservation in time. Moreover, in this experiment we compared two different sizes of particles (74–420 µm and 500–1700 µm) while the rhBMP6 dose was 125 µg/per implant according to the findings of the first experiment. Different Osteogrow-C formulations were implanted on the left and right side to reduce the biological variability. In brief, in the first experiment Osteogrow C implants containing either TCP and BCP were implanted in each animal, while applied rhBMP6 dose was different on the left (125 µg) and right (250 µg) side. In the second experiments each animal received Osteogrow C implants with either TCP or BCP while size of ceramic particles differed on different sides (500–1700 µm particles were implanted on the left side and 74–420 µm particles were implanted on the right side) The number of implants was four per group in the first and six per group in the second experiment. The sample size was determined based on previous studies in this model and recommendations on the rational use of experimental animals including 3R principle (replacement, reduction, refinement). Experimental design of the study is presented in Table 1. Results were retrospectively compared with our previously published results in rabbit PLF 7 weeks after surgery using modified and unpublished results^37^. In the published study, we used Osteogrow C implants containing 125 µg rhBMP6 in ABC with 500–1700 µm TCP or BCP (TCP/HA in 80/20 ratio) ceramic particles and therefore results of this experiment were comparable with analog formulations evaluated in the present study.

**Table 1:** Rabbit PLF study design with 14 and 27 weeks follow up.

**Observation period 14 weeks (n = 4 per group)**	
A	TCP 500–1700 µm + 125 µg BMP6 in ABC
B	TCP 500–1700 µm + 250 µg BMP6 in ABC
C	BCP (TCP/HA 80/20) 500–1700 µm + 125 µg BMP6 in ABC
D	BCP (TCP/HA 80/20) 500–1700 µm + 250 µg BMP6 in ABC
**Observation period 27 weeks (n = 6 per group)**	
A	TCP 74–420 µm + 125 µg BMP6 in ABC
B	BCP (TCP/HA 40/60) 74–420 µm + 125 µg BMP6 in ABC
C	TCP 500–1700 µm + 125 µg BMP6 in ABC
D	BCP (TCP/HA 40/60) 500–1700 µm + 125 µg BMP6 in ABC

### Experimental animals

Experiments were conducted in 20 New Zealand White rabbits (lat. *Oryctolagus cuniculus*, 20-week-old, male, the bodyweight 3–5 kg), housed in standard rabbit cages with environmental enrichment in conventional laboratory conditions (temperature 18–24 °C, relative humidity 50–70%, noise level 60 dB and illumination 12 h/day) at the registered animal facility of Laboratory for Mineralized Tissues (HR-POK-001). Standard diet and freshwater were provided ad libitum. Approval for the studies was given by the Directorate for Veterinary and Food Safety, Ministry of Agriculture, Republic of Croatia. Ethical principles of the study ensured compliance with European Directive 2010/63/EU, the Law on amendments to animal protection act (Official Gazette 37/13), the Animal Protection Act (Official Gazette 102/17), Ordinance on the protection of animals used for scientific purposes (Official Gazette 55/13), ARRIVE guidelines, FELASA recommendations, and recommendations of the Ethics Committee at School of Medicine, University of Zagreb and National Ethics Committee (EP 187/2018).

### ABGS preparation

**Figure 8:**
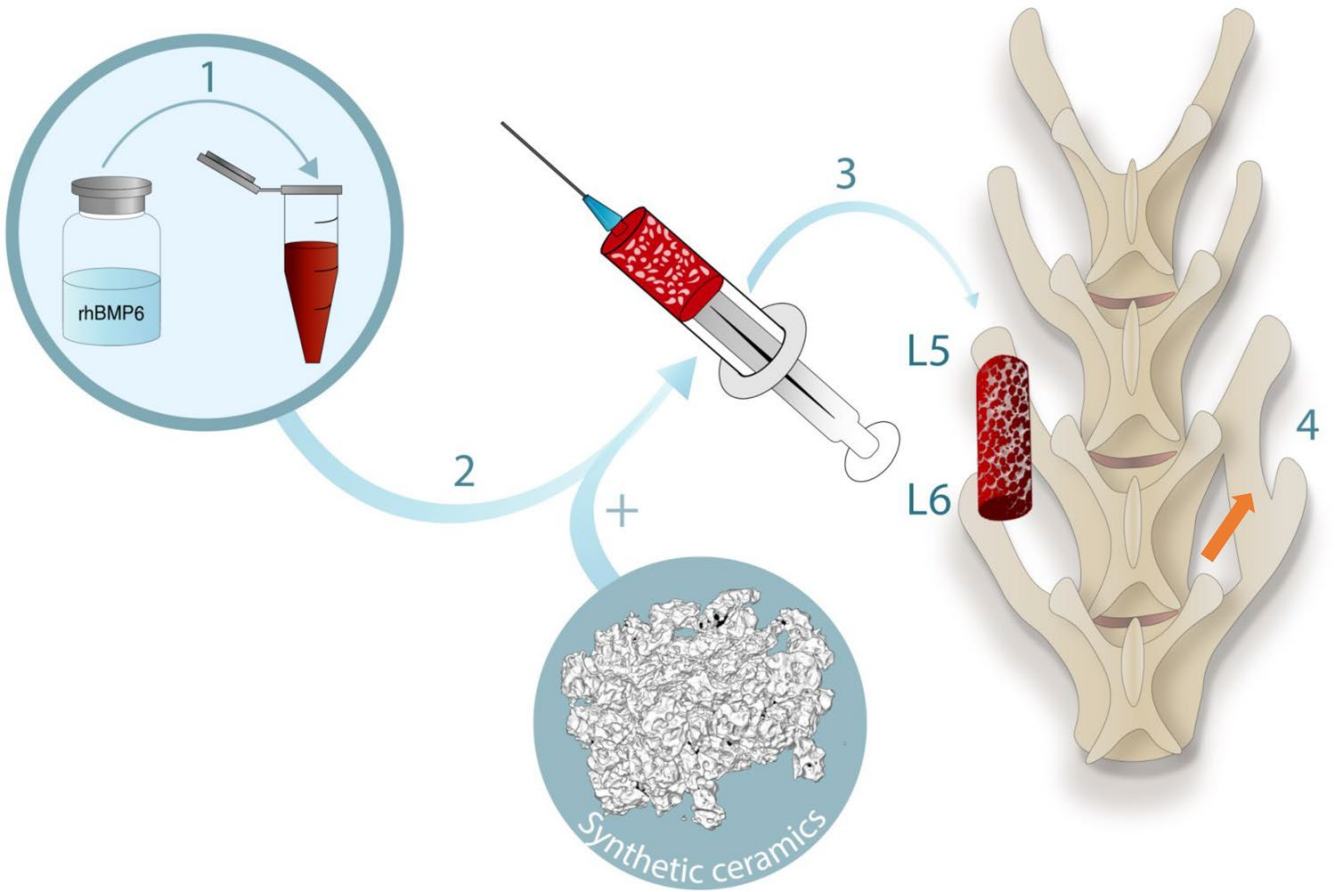
ABGS (Osteogrow-C) preparation and implantation. (1) rhBMP6 was dissolved in water for injection and added to blood withdrawn from the marginal rabbit ear vein. Blood containing rhBMP6 was mixed with ceramic particles placed in a syringe (2) and left at room temperature to coagulate for 60–90 min. ABGS implant was detached from the syringe wall, placed in a Petri dish and implanted in the lateral gutter between L5-L6 transverse processes (3). At the end of the follow-up period the newly formed bone induced by ABGS bridged the gap between transverse processes (orange arrow) (4).

Autologous blood (2.5 mL per implant) was collected from the rabbit marginal ear vein into tubes without an anticoagulant substance containing rhBMP6 (125 or 250 µg according to the experimental design) as previously described^34,37^. Blood was drawn into syringes (5 mL) containing ceramic particles (0.5 g of 500–1700 μm particles or 0.8 g of 74–420 μm particles), gently mixed, and rotated until blood coagulated to achieve uniform particle distribution in the implant (Fig. 8). The amount of 74–420 µm particles was higher than 500–1700 µm particles to achieve the same volume of graft material (1 cc) and subsequently uniform distribution of particles in the implant. The final rhBMP6 concentration in the implants was 50 and 100 µg/ml for implants containing 125 and 250 µg of rhBMP6, respectively. Following blood coagulation, implants were stored at 4 °C and implanted within 2 h after preparation according to the rhBMP6/ABC stability determined in Osteogrow preclinical studies.

### Surgical procedure

Twenty New Zealand White rabbits underwent bilateral posterolateral lumbar fusion (L5-L6) as described^34,37^. Animals were premedicated with a mixture of Xylazine (Xylapan^®^, Vetoquinol, Switzerland) 5 mg/kg and Ketamine (Ketaminol^®^ Vetoquinol, Switzerland) 35 mg/kg applied intramuscularly. General anesthesia was maintained using a mixture of isoflurane (1–1.5%) and oxygen delivered by mask. A dorsal midline skin incision extending from L4 to L7 was made, followed by a paramedian fascial incision. Paravertebral muscles were retracted laterally, facilitating exposure of L5–L6 transverse processes. Exposed transverse processes were then decorticated using a high-speed burr, and Osteogrow-C implants were placed bilaterally in the gutter between L5–L6 transverse processes (Fig. 8). The fascial and skin incisions were closed with 3‐0 synthetic glycolide/lactide copolymer absorbable sutures in a continuous fashion. There were no adverse effects during the follow-up period. Experimental animals were euthanized 14 or 27 weeks following Osteogrow-C implantation using premedication of 3 mg/kg xylazine and 20 mg/kg ketamine i.m. and administration of T61 (1 ml/kg) i.v. Rabbit lumbar spine was harvested to conduct analyses described in the following sections.

### Segmental mobility testing

The success of spinal fusion was preliminary determined by manual palpation. Spinal fusion was considered succesful if upon manual compression observed spinal segment (L5–L6) was immobile and without any motion regularly observed between the vertebrae. The manual palpation test was conducted by two independent researchers (N.S. and N.I.).

### MicroCT analyses

MicroCT analyses were used to evaluate the success of spinal fusion, osseointegration of newly formed bone with adjacent transverse processes, and to determine the amount of newly formed bone and CRM among experimental groups. At the end of the experiment, lumbar spine of all animals was scanned with 1076 SkyScan MicroCT (Bruker, Belgium) as described^37,60^. In brief, scanning resolution was set at 18 µm, frame averaging was set to a value of 2, and a 0.5 mm aluminum filter was used. Following the acquisition, images were reconstructed using NRecon software (Bruker, Belgium) and analyzed by CTAn software (Bruker, Belgium).

### Histology

All specimens were fixed in 10% neutral buffered formalin. Following biomechanical testing, three or four samples per group were decalcified using 14% EDTA in 4% formalin solution (20 days) for histological analysed. Following decalcification specimens were embedded in paraffin, cut at 6 µm section and stained with Goldner's trichrome stain as described^56^. The rest of the specimens (one or two per group) were processed undecalcified as previously described^37^. In brief, specimens were dehydrated, cleared manually with methyl salicylate and xylenes. Specimens were polymerized into hardened acrylic resin blocks (MMA), and 5 µm microtomed sections were obtained using tungsten-carbide knives (D-profile, Delaware Diamond Knives, Delaware, USA) and an automated sled microtome (SM2500, Leica Biosystems, Illinois, USA). Finally, sections were mounted on gelatin-coated glass microscope slides, unplasticized, hydrated, and stained with Von Kossa and Goldner's trichrome stain.

### Histomorphometry

Histomorphometric analyses were conducted to analyze the architecture of newly formed bone and to determine the area occupied by bone, bone marrow, and ceramic particles. The region of interest for histomorphometrical analyses was the newly formed bone between transverse processes. In the analysis we excluded newly formed bone adjacent/superpositioned to transverse processes since it was difficult to distinguish between the new and native bone. Quantitative analyses were conducted on histological sections stained by Goldner, and images were taken using an Olympus BX53 Upright Microscope equipped with a DP27 camera (5 megapixels, 15 fps) and operated by cellSens Dimension software (Olympus, Japan). The Photoshop software (Adobe Systems, California, USA) was used to select and mask areas of interest (bone and ceramic particles) with the distinctive color, and Fiji ImageJ software (NIH, Maryland, USA) was used to measure the masked areas as described before^37,56^. Results were expressed as the area percentage.

### Biomechanical testing

Biomechanical parameters (maximum force, elasticity, and work-to-break) of newly formed bone integrated with adjacent transverse processes were determined by a three-point bending test using TA.HDplus instrument (Stable Micro Systems, UK). The fusion mass was placed on two supports, and the force was applied perpendicular to the midpoint as previously described^37^.

### Data analysis

Kolmogorov–Smirnov test was used to test whether the data follows Gaussian or non-Gaussian distribution. Gaussian-distributed data was analysed using unpaired t-test (two experimental groups) or ordinary one-way ANOVA with Tukey's multiple comparisons tests (three or more experimental groups). Non-Gaussian distributed data was analysed with Mann–Whitney U test (two experimental groups) or Kruskal–Wallis test with Dunn's multiple comparisons test (three or more experimental groups). Data are shown as mean with standard deviation (SD) or as median with a range of minimum and maximum values. Significant P (P < 0.05) values are marked with asterisks; *(P ≤ 0.05), **(P ≤ 0.01), ***(P ≤ 0.001). In all statistical analyses statistical software GraphPad Prism (v.8.4.3) was used.

## Data Availability

Raw data were generated at the Laboratory for Mineralized Tissues. Derived data supporting the findings of this study are available from the corresponding author, S.V., upon request.
